# Therapeutic Potential of the Hsp90/Cdc37 Interaction in Neurodegenerative Diseases

**DOI:** 10.3389/fnins.2019.01263

**Published:** 2019-11-21

**Authors:** Liam Gracia, Gabriella Lora, Laura J. Blair, Umesh K. Jinwal

**Affiliations:** ^1^Department of Pharmaceutical Sciences, Taneja College of Pharmacy, University of South Florida-Health, Tampa, FL, United States; ^2^Department of Molecular Medicine, Byrd Alzheimer’s Institute, Morsani College of Medicine, University of South Florida, Tampa, FL, United States

**Keywords:** Hsp90, chaperone, Cdc37, kinase, Alzheimer’s, Huntington’s, Parkinson’s

## Abstract

Alzheimer’s, Huntington’s, and Parkinson’s are devastating neurodegenerative diseases that are prevalent in the aging population. Patient care costs continue to rise each year, because there is currently no cure or disease modifying treatments for these diseases. Numerous efforts have been made to understand the molecular interactions governing the disease development. These efforts have revealed that the phosphorylation of proteins by kinases may play a critical role in the aggregation of disease-associated proteins, which is thought to contribute to neurodegeneration. Interestingly, a molecular chaperone complex consisting of the 90 kDa heat shock protein (Hsp90) and Cell Division Cycle 37 (Cdc37) has been shown to regulate the maturation of many of these kinases as well as regulate some disease-associated proteins directly. Thus, the Hsp90/Cdc37 complex may represent a potential drug target for regulating proteins linked to neurodegenerative diseases, through both direct and indirect interactions. Herein, we discuss the broad understanding of many Hsp90/Cdc37 pathways and how this protein complex may be a useful target to regulate the progression of neurodegenerative disease.

## Introduction

Molecular chaperones are important regulators of cellular homeostasis (Csermely et al., 1998; Buchner, 2013; Balchin et al., 2016; Lackie et al., 2017; Rosenzweig et al., 2019). The major chaperone, heat shock protein 90 (Hsp90), is a highly abundant and conserved protein, comprising of roughly 1–2% of the total proteome (Csermely et al., 1998; Crevel et al., 2001). Hsp90, like other Hsp family members, interacts directly with protein clients to maintain cellular organization (Csermely et al., 1998; Buchner, 2013; Lackie et al., 2017). In particular, Hsp90 assists with the final maturation steps of client proteins, such as kinases (Millson et al., 2005; Vaughan et al., 2008).

In mammalian cells, Hsp90 requires partner proteins, termed co-chaperones, to assist with client triage (Smith, 1993; Sahasrabudhe et al., 2017). One of the well-studied Hsp90 co-chaperones is Cell Division Cycle 37 (Cdc37) (Dey et al., 1996; Stepanova et al., 1996; MacLean and Picard, 2003). The Hsp90/Cdc37 complex interacts with approximately 60% of the kinome (Eckl et al., 2015; Luo et al., 2017; Verba and Agard, 2017). Cdc37, a signal transducer, binds to a diverse set of kinase clients (Verba and Agard, 2017). Independent actions of Cdc37 have also been described. For example, the stability of a discrete Cyclin-dependent kinase 4 (CDK4) was reported to be dependent on Cdc37 levels, but not on the interaction of Cdc37 with Hsp90 (Smith et al., 2015).

Considerable efforts have been made toward defining the interactions of Hsp90 kinase co-chaperone Cdc37, but the majority of these studies have been performed in cancer models (Calderwood, 2015; Li D. et al., 2015; Wang et al., 2017). However, many kinases that are regulated by Cdc37 have also been implicated in neurodegenerative disease-related pathways (Branca et al., 2017; Kirouac et al., 2017; Lackie et al., 2017; Lazarevic et al., 2017; Li and Götz, 2017; Yang et al., 2018; Zhang et al., 2018). Hsp90 has been suggested as a therapeutic target for these disorders, but Hsp90 inhibitors have faced many challenges in the clinic (Neckers and Workman, 2012; Neckers et al., 2018). Targeting Hsp90 co-chaperones, such as Cdc37, may represent an alternative strategy (Moses et al., 2018; Sluder et al., 2018). Here, we describe the evidence supporting a role of Cdc37 as a molecular target in neurological disorders, such as Alzheimer’s (AD), Parkinson’s (PD), and Huntington’s (HD) disease.

## The Hsp90/Cdc37 Complex Regulates Kinases

Kinases are critical regulators of cell cycle progression, signal transduction, and transcription regulation (Liu et al., 1999; Taylor et al., 2012; Verba and Agard, 2017). Therefore, proteins that regulate kinases, such as Hsp90, have broad implications in disease pathogenesis. Hsp90 functions as a homodimer, through the C-terminal domain (CTD) dimerization, and is primarily characterized by its ATPase activity in the N-terminal domain (NTD) (Siligardi et al., 2002; Zhang et al., 2015). Kinase interaction with Hsp90 generally promotes kinase stabilization and activity (Luo et al., 2017; Bachman et al., 2018). The Hsp90 co-chaperone, Cdc37, is a ubiquitous protein that is required for efficient Hsp90-mediated maturation of kinases, such as CDK5, extracellular regulated kinase (ERK), and protein kinase B (Akt), by aiding their partnership with Hsp90 (Dey et al., 1996; Stepanova et al., 1996; Jinwal et al., 2011; Smith et al., 2015; Keramisanou et al., 2016; Wang et al., 2016; Verba and Agard, 2017; Liu et al., 2018). Strong Hsp90-Cdc37 binders have been characterized to be less thermodynamically stable on their own, whereas clients that are more stable have reduced dependence on Hsp90/Cdc37 for maturation (Taipale et al., 2012; Verba and Agard, 2017). During this interaction, Cdc37 binds clients using NTD residues, the middle domain of Cdc37 then associates with the NTD of Hsp90, and, finally, ATP binding causes Cdc37 to transition into the middle domain of Hsp90 to promote kinase changes (MacLean and Picard, 2003; Verba et al., 2016; Verba and Agard, 2017). Additional roles for the Hsp90/Cdc37 complex have also been described. In fact, previous studies have demonstrated that the shuttling of kinases to Hsp90 can sheathe proteins from further activation or ubiquitination (Citri et al., 2004; Ota and Wang, 2012). Recent reports also suggest that the Hsp90/Cdc37 interaction is important in preventing activated kinase aggregation (Tripathi et al., 2014; Verba and Agard, 2017). Additionally, it should be noted that clients can be regulated through augmentation of Hsp90’s C-terminal MEEVD domain (Prodromou et al., 1997; Siligardi et al., 2002; Verba and Agard, 2017). This region is known to interact with tetratricopeptide repeat domain containing co-chaperones, which can further affect client binding and regulation (Oberoi et al., 2016; Verba and Agard, 2017). One of these co-chaperones, the serine/threonine protein phosphatase 5 (PP5), has been shown to also regulate the activity of Cdc37 by altering its phosphorylation status at serine 13 (Vaughan et al., 2008). Related to this, casein kinase 1 (Dushukyan et al., 2017) and casein kinase 2 (Mollapour et al., 2011) have been shown to alter Cdc37 phosphorylation through PP5 activation and through affecting the Hsp90/Cdc37 interaction, respectively. Interestingly, these casein kinases have also been implicated to have involvement in the pathogenesis of neurodegenerative diseases, including AD and PD (Perez et al., 2011).

While there are no small molecules that specifically target Cdc37 or the Hsp90/Cdc37 complex, both Celastrol and Withaferin A have been shown to be capable of disrupting the Hsp90/Cdc37 complex, along with targeting other pathways in the cell (Zhang and Sarge, 2007; Yu et al., 2010). Celastrol can disrupt recombinant Hsp90/Cdc37 interaction at 50–100 μM concentrations (Zhang et al., 2009), while another study in a cell-based model showed 12.5 μM is sufficient (Peng et al., 2016). These studies suggest that the concentrations of Celastrol required to disrupt Hsp90/Cdc37 may vary between different model systems. It is also known that Celastrol at nanomolar dose shown to suppress the production of proinflammatory cytokines TNF-α and IL-1ß and improve cognitive processes (Allison et al., 2001). Celastrol has also been reported to affect Hsp90 through alternative mechanisms, including activation of the heat shock response (Westerheide et al., 2004), indirectly through redox imbalance (Seo et al., 2011; Klaić et al., 2012), and, recently, Celastrol was shown to induce Hsp90 oligomerization (Zanphorlin et al., 2014). Celastrol is also capable of binding and triggering oligomerization of p23, an Hsp90 co-chaperone (Chadli et al., 2010). Withaferin A is mostly studied for its anti-inflammatory properties (Misra et al., 2008). Thus, the effects of Celastrol and Withaferin A cannot be assumed to be only through Hsp90 and Cdc37, but instead may be partially mediated by the disruption of this complex. More recently, additional compounds have been reported including, Kongensis A, a possible necroptosis and inflammation inhibitor that can bind to Hsp90 and dissociate its interaction with Cdc37 (Li et al., 2016), and platycodin D, which has anticancer and immune regulatory properties that was also shown to disrupt the Hsp90/Cdc37 complex (Li et al., 2017). However, these drugs have not yet been tested in models of neurodegeneration.

### Alzheimer’s Disease

Alzheimer’s disease is a progressive neurodegenerative disease and the most common form of dementia (Masters et al., 2015; Bondi et al., 2017). While the cause of AD is still not completely clear (Davis et al., 2018; Shi and Holtzman, 2018; Henstridge et al., 2019), AD is hallmarked by the accumulation of protein aggregates, most notably plaques between neurons, rich in β-amyloid (Aβ), and intraneuronal tangles, composed mainly of tau protein. The aggregation of these proteins is not the only cause of AD, but they are each thought to contribute to the progression. In fact, there are numerous pathways implicated in the pathogenesis of AD, including multiple kinases that may promote or stabilize amyloid accumulation (Martin et al., 2013; Li X. et al., 2015; Ahmad et al., 2018). Aβ can be affected by kinases directly (Rezaei-Ghaleh et al., 2016) and also indirectly through the phosphorylation of the amyloid precursor protein (APP) (Chang et al., 2006), which can alter the cleavage dynamics and Aβ generation. In fact, multiple kinases have been linked to Aβ plaque production or stability including: CDK5, dual-specificity tyrosine phosphorylation-regulated kinase 1A (DYRK1A), Akt, ERK, Fyn, c-Abl, and glycogen synthase kinase 3α (GSK3α) (Phiel et al., 2003; Chin et al., 2004, 2005; Cancino et al., 2011; Castro-Alvarez et al., 2015; Coutadeur et al., 2015; Park et al., 2015; Estrada et al., 2016; Branca et al., 2017; Kirouac et al., 2017; Lazarevic et al., 2017; Li and Götz, 2017; Yang et al., 2018; Zhang et al., 2018). Some of these kinases, for example, c-Abl, have been shown to alter Hsp90 phosphorylation as well as affect other Hsp90 co-chaperones, like Activator of Hsp90 ATPase protein 1 (Aha1) (Dunn et al., 2015). Thus, the interactions between kinases, chaperones, and substrates is quite complex. Additionally, many of these kinases have been shown to be either regulated by Cdc37 (Gray et al., 2007; Jinwal et al., 2011; Sonamoto et al., 2015) and/or be a Cdc37 interactor (Taipale et al., 2012). Interestingly, even though Hsp90 inhibition shows protection in Aβ models (Chen et al., 2014; Ortega et al., 2014), studies investigating the role of Cdc37 in Aβ pathogenesis have not been reported. However, Cdc37 is likely to at least have an indirect role on Aβ deposition and stability through the regulation of these important kinases.

Kinases are strongly implicated in tau pathogenesis, as tau is found in a hyperphosphorylated state in the AD brain (Cavallini et al., 2013; Simic et al., 2016). These kinases include: CDK5, DYRK1A, Akt, ERK/mitogen-activated protein kinase (MAPK), Fyn, c-Abl, GSK3β, protein kinase C (PKC), and the microtubule affinity regulating kinase 2 (MARK2), as well as Cdc37 (Jayapalan and Natarajan, 2013; Mietelska-Porowska et al., 2014; Calderwood, 2015; Wang et al., 2015, 2016; Bondi et al., 2017; Callender and Newton, 2017; Kang et al., 2017; Lee and Kim, 2017; Li and Götz, 2017; Liu et al., 2017; Seo et al., 2017). Data from our group demonstrated that Cdc37 in collaboration with Hsp90 stabilized tau, and, inversely, reduced Cdc37 promoted tau clearance (Jinwal et al., 2011). These effects on tau are likely a combination of direct interactions as well as indirect effects on other kinases. In fact, CDK5 and Akt, which can also promote tau phosphorylation (Jayapalan and Natarajan, 2013; Callender and Newton, 2017; Liu et al., 2017; Seo et al., 2017), are stabilized by Cdc37 (Jinwal et al., 2011). Akt activity inversely regulates GSK3β, which also phosphorylates tau (Jiang et al., 2015; Wang et al., 2015, 2016; Kang et al., 2017), but other pathways can also regulate GSK3β (Hermida et al., 2017). Interestingly, GSK3β was recently reported to bind the Hsp90/Cdc37 complex (Jin et al., 2016).

Prior work from our group demonstrated that Cdc37 modulation did not robustly affect GSK3β stability *in vitro* (Jinwal et al., 2011). MARK2, another kinase known to regulate tau, displayed a similar lack of effect following Cdc37 regulation. DYRK1A, Fyn, and c-Abl have been implicated in a number of studies to regulate tau phosphorylation (Ryoo et al., 2007; Coutadeur et al., 2015; Lau et al., 2016; Yin et al., 2017). All of these kinases have been shown to bind and/or be regulated by Cdc37 (Yun and Matts, 2005; Tsukahara and Maru, 2010; Taipale et al., 2012; Sonamoto et al., 2015), however, the synergy between these proteins on tau regulation has not been investigated. Cdc37 may also affect tau phosphorylation through the regulation of MAPK and PKC (Gray et al., 2007; Gould et al., 2009), but the contribution of these kinases on tau phosphorylation is still under investigation. Some reports suggest that ERK activation, which is downstream of via MAPK, alters tau phosphorylation (Lee and Kim, 2017; Li and Götz, 2017), while others have shown no effect (Noël et al., 2015). In many models, PKC has been reported to phosphorylate GSK3β, thereby inhibiting its ability to phosphorylate tau residues (Correas et al., 1992; Isagawa et al., 2000; De Montigny et al., 2013); however, PKC can directly phosphorylate tau residues (Calderwood, 2015).

Taken together, these data suggest that Cdc37 regulation can directly alter tau phosphorylation and stability as well as indirectly affect tau through other kinases, such as Akt, CDK5, but shows minimal effects on the regulation of GSK3β, MARK2, while the collaborative effects of Cdc37 with MAPK, PKC, DYRK1A, Fyn, and c-Abl on tau have yet to be determined. Celastrol reduced tau phosphorylation by inhibiting Hsp90 (Cao et al., 2018), which may be partially mediated by Cdc37 disruption. Overall, Cdc37 can regulate several pathways implicated in AD pathogenesis, including multiple that affect both Aβ and tau.

### Parkinson’s Disease

The second most prevalent neurodegenerative disease is PD, which involves a decline in motor as well as cognitive abilities (Saiki et al., 2012; Hayes, 2019). The cause of PD is not known, but has been primarily linked to a decline in mitochondrial function, oxidative stress, dysregulation of multiple signaling pathways, and the formation of Lewy bodies (Schapira and Jenner, 2011). Lewy bodies primarily consist of misfolded α-synuclein. It has been suggested by our previous work that Cdc37 does not globally alter α-synuclein stability (Jinwal et al., 2011), but instead may contribute to the regulation of α-synuclein phosphorylation, which is highly linked to its aggregation. It is unknown if Cdc37 directly alters α-synuclein phosphorylation, but likely affects known α-synuclein regulating kinases, which include the Src family of kinases (including Lck and Fyn), casein kinase, and G protein-coupled receptor kinases (Kimura et al., 1997; Luo and Benovic, 2003; Nika et al., 2010; Taipale et al., 2012). In addition, Cdc37 may affect other pathways linked to PD pathogenesis.

Mitochondrial dysfunction has been strongly linked to PD (Park et al., 2018). In fact, hereditary PD genes encode mitochondrial proteins (Schapira and Jenner, 2011; Lill, 2016). For example, the Parkin (PRKN)/PTEN-induced kinase 1 (Pink1) pathway, which is essential for mitochondrial quality control (Saiki et al., 2012). Interestingly, several studies have demonstrated interaction with Hsp90/Cdc37 may be critical in the regulation of Pink1, by regulating Pink1 stability (Weihofen et al., 2007; Moriwaki et al., 2008; Baldo et al., 2012). Hsp90/Cdc37 interaction is also crucial for proper Pink1 processing and subcellular localization (Weihofen et al., 2007). However, contrary data has also been reported, which shows a PD-associated mutant Pink1 isoform is degraded too quickly and fails to bind Hsp90/Cdc37 (Moriwaki et al., 2008).

Mutations in the leucine-rich repeat kinase 2 (LRRK2) gene cause PD (Gandhi et al., 2009; Cookson, 2010). Although the function of LRRK2 is unknown, its kinase activity has been demonstrated to activate multiple signaling pathways that suggest it contributes to neurite outgrowth and cytoskeletal maintenance (Gandhi et al., 2009; Cookson, 2010). Therefore, abrogating aberrant LRRK2 kinase activity is of interest in PD (Alessi and Sammler, 2018). Hsp90 inhibitors have been shown to be effective at preventing LRRK2 toxic gain of function, by disrupting LRRK2 from the Hsp90/Cdc37 complex (Wang et al., 2008). Withaferin A can also reduce LRRK2 levels, in part through inhibiting the Hsp90/Cdc37 interaction (Narayan et al., 2015). Based on these evidences, the Cdc37/Hsp90 complex may be a reasonable target for drug discovery in PD.

### Huntington’s Disease

Huntington’s disease is an inherited neurodegenerative disease that severely impacts motor function and often impairs cognition (La Spada et al., 2011; Caron et al., 2018), which is caused by an autosomal dominant mutation in the huntingtin gene that gives rise to a CAG trinucleotide repeat expansion (Schulte and Littleton, 2011). This generates cytoplasmic and nuclear protein aggregates that cause disturbances of the cellular proteasome affecting several pathways and ultimately resulting in neurotoxicity (Rosenstock et al., 2012; Caron et al., 2018). Hsp90 can interact with huntingtin protein (Baldo et al., 2012), and inhibition of Hsp90 can block mutant huntingtin aggregation through inducing the heat shock response (Sittler et al., 2001). Celastrol can also inhibit mutant huntingtin aggregation by a similar mechanism (Zhang and Sarge, 2007).

Similar to tau, Akt, which is altered in HD brain, can directly phosphorylate mutant huntingtin protein, which can protect against aggregation and neuronal toxicity (Humbert et al., 2002). Downregulation of Akt has also been shown in a HD animal model (Colin et al., 2005). As mentioned above, the Hsp90/Cdc37 complex can impact Akt stability (Basso et al., 2002). Another pathway of interest in HD pathogenesis with relation to Cdc37 is the I kappa B kinase (IKK)/nuclear factor kappa-light-chain-enhancer (NFkB) inflammatory response, which can be chronically upregulated in HD (Rosenstock et al., 2012; Bowles and Jones, 2014). The IKK kinase complex is responsible for activating the NFKB transcription factor, which triggers expression of the inflammatory genes. Recruitment of Cdc37 to Hsp90 is required for proper IKK catalytic activation (Hinz et al., 2007). Interestingly, IKK inhibitors show neuroprotection in a brain slice HD model (Reinhart et al., 2011). Celastrol can inhibit IKK (Lee and Kim, 2017), which is suggested to be a direct action, but it is possible that disruption of the Cdc37/Hsp90 complex may also contribute (Zhang et al., 2009). Overall, these evidences suggest the Hsp90/Cdc37 complex deserves further investigation as a therapeutic target in HD.

## Final Words

**FIGURE 1:**
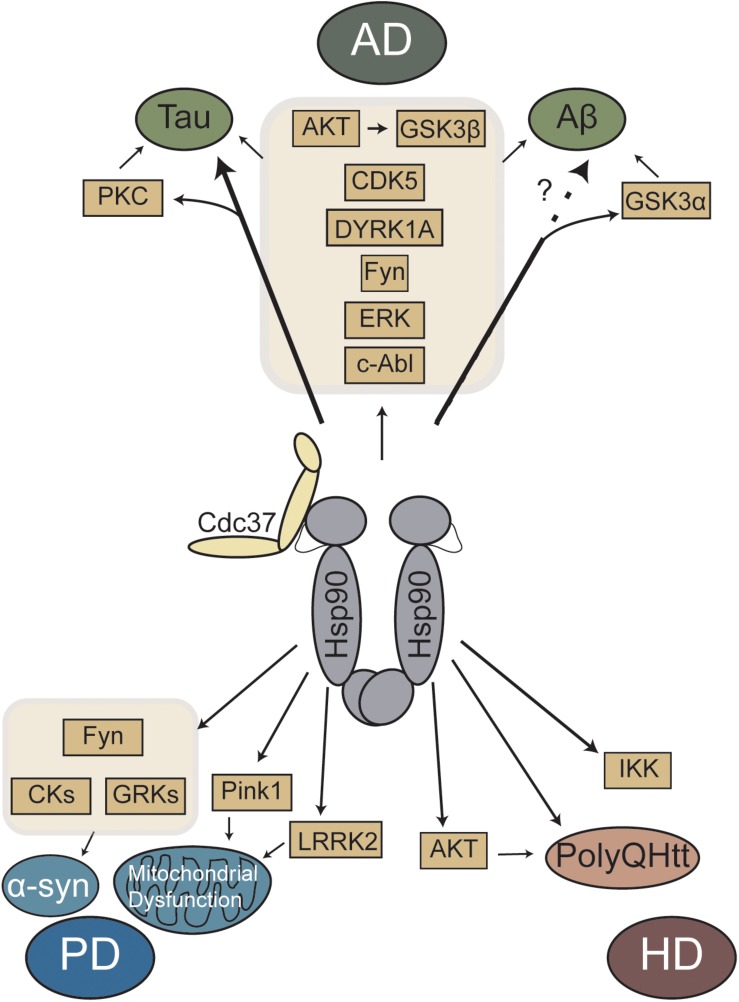
Schematic representation of Hsp90/Cdc37 regulated major kinases and proteins linked to neurodegenerative diseases. AD, Alzheimer’s disease; PD, Parkinson’s disease; HD, Huntington’s disease.

Overall, Hsp90 and Cdc37 regulates many neurodegenerative disease-linked proteins (Figure 1). Experimental evidence suggests that client proteins may be differentially affected based on their strength of Hsp90/Cdc37 binding, which may allow for targeting a specific subset of clients without affecting the whole pool. Recent work has started to characterize these interactions (Verba and Agard, 2017), but additional studies are needed to better clarify these differences and how they are affected in disease. The ability to regulate many pathways through a single protein complex is exciting, but it is unlikely that any single protein complex will be the sole solution for any neurodegenerative disease. These diseases are very complex and may be more of a spectrum than individual disorders (Villemagne et al., 2015). However, additional research is still needed to better understand the benefits of targeting Cdc37, as well as other exciting targets, in these diseases. It is also important to note that there are other neurodegenerative diseases that were not discussed in this review, such as amyotrophic lateral sclerosis (ALS). In ALS, there are some unique pathways as well as many overlapping with those diseases discussed, including data to suggest the Hsp90/Cdc37 pathway (Jinwal et al., 2012). While the effects of Hsp90 inhibitors in neurodegenerative models of disease has been tested, the investigation of targeting Hsp90/Cdc37 is still underexplored. Celastrol and Withaferin A, along with Hsp90 inhibitors, like 17-AAG, albeit not specific to Cdc37, can regulate this interaction. Perhaps with the development of the next phase of Hsp90 inhibitors more tools will be available to target this complex (Neckers et al., 2018). In addition, the recent discovery of novel drugs that regulate the Hsp90/Cdc37 interaction, like platycodin D and Kongensis A (Li et al., 2016, 2017), provides new avenues for investigation.

## Author Contributions

LG and GL equally contributed to write this review with support from UJ and LB. UJ supervised the project.

## Conflict of Interest

The authors declare that the research was conducted in the absence of any commercial or financial relationships that could be construed as a potential conflict of interest.

## References

[B1] Ahmad S. Bannister C. Van Der Lee S. J. Vojinovic D. Adams H. H. H. Ramirez A. (2018). Disentangling the biological pathways involved in early features of Alzheimer’s disease in the Rotterdam Study. *Alzheimers Dement* 14 848–857. 10.1016/j.jalz.2018.01.005 29494809

[B2] Alessi D. R. Sammler E. (2018). LRRK2 kinase in Parkinson’s disease. *Science* 360 36–37. 29622645 10.1126/science.aar5683

[B3] Allison A. C. Cacabelos R. Lombardi V. R. M. Álvarez X. A. Vigo C. (2001). Celastrol, a potent antioxidant and anti-inflammatory drug, as a possible treatment for Alzheimer’s disease. *Prog. Neuro. Psychopharmacol. Biol. Psychiatr.* 25 1341–1357. 10.1016/s0278-5846(01)00192-0 11513350

[B4] Bachman A. B. Keramisanou D. Xu W. Beebe K. Moses M. A. Vasantha Kumar M. V. (2018). Phosphorylation induced cochaperone unfolding promotes kinase recruitment and client class-specific Hsp90 phosphorylation. *Nat. Commun.* 9 265–265. 10.1038/s41467-017-02711-w 29343704 PMC5772613

[B5] Balchin D. Hayer-Hartl M. Hartl F. U. (2016). In vivo aspects of protein folding and quality control. *Science* 353:aac4354. 10.1126/science.aac4354 27365453

[B6] Baldo B. Weiss A. Parker C. N. Bibel M. Paganetti P. Kaupmann K. (2012). A screen for enhancers of clearance identifies huntingtin as a heat shock protein 90 (Hsp90) client protein. *J. Biol. Chem.* 287 1406–1414. 10.1074/jbc.M111.294801 22123826 PMC3256905

[B7] Basso A. D. Solit D. B. Chiosis G. Giri B. Tsichlis P. Rosen N. (2002). Akt Forms an Intracellular complex with heat shock protein 90 (Hsp90) and Cdc37 and is destabilized by inhibitors of Hsp90 function. *J. Biol. Chem.* 277 39858–39866. 10.1074/jbc.m206322200 12176997

[B8] Bondi M. W. Edmonds E. C. Salmon D. P. (2017). Alzheimer’s disease: past, present, and future. *J. Int. Neuropsychol. Soc.* 23 818–831. 10.1017/S135561771700100X 29198280 PMC5830188

[B9] Bowles K. Jones L. (2014). Kinase signalling in Huntington’s disease. *J. Huntingtons Dis.* 3 89–123. 10.3233/JHD-140106 25062854

[B10] Branca C. Shaw D. M. Belfiore R. Gokhale V. Shaw A. Y. Foley C. (2017). Dyrk1 inhibition improves Alzheimer’s disease-like pathology. *Aging Cell* 16 1146–1154. 10.1111/acel.12648 28779511 PMC5595697

[B11] Buchner J. L. J. (2013). Structure, function and regulation of the hsp90 machinery. *Biomed. J.* 36 106–117. 10.4103/2319-4170.113230 23806880

[B12] Calderwood S. K. (2015). “Cdc37 as a Co-chaperone to Hsp90,” in *The Networking of Chaperones by Co-Chaperones: Control of Cellular Protein Homeostasis*, eds Blatch G. L. Edkins A. L. (Cham: Springer International Publishing), 103–112. 10.1007/978-3-319-11731-7_5

[B13] Callender J. A. Newton A. C. (2017). Conventional protein kinase C in the brain: 40 years later. *Neuro. Signal.* 1:NS20160005. 10.1042/NS20160005 PMC7373245 32714576

[B14] Cancino G. I. Perez De Arce K. Castro P. U. Toledo E. M. Von Bernhardi R. Alvarez A. R. (2011). c-Abl tyrosine kinase modulates tau pathology and Cdk5 phosphorylation in AD transgenic mice. *Neurobiol. Aging* 32 1249–1261. 10.1016/j.neurobiolaging.2009.07.007 19700222

[B15] Cao F. Wang Y. Peng B. Zhang X. Zhang D. Xu L. (2018). Effects of celastrol on Tau hyperphosphorylation and expression of HSF-1 and HSP70 in SH-SY5Y neuroblastoma cells induced by amyloid-β peptides. *Biotechnol. Appl. Biochem.* 65 390–396. 10.1002/bab.1633 29274099

[B16] Caron N. S. Dorsey E. R. Hayden M. R. (2018). Therapeutic approaches to huntington disease: from the bench to the clinic. *Nat. Rev. Drug Discov.* 17:729. 10.1038/nrd.2018.133 30237454

[B17] Castro-Alvarez J. F. Uribe-Arias A. Cardona-Gómez G. P. (2015). Cyclin-Dependent kinase 5 targeting prevents β-Amyloid aggregation involving glycogen synthase kinase 3β and phosphatases. *J. Neurosci. Res.* 93 1258–1266. 10.1002/jnr.23576 25711385 PMC4478163

[B18] Cavallini A. Brewerton S. Bell A. Sargent S. Glover S. Hardy C. (2013). An unbiased approach to identifying tau kinases that phosphorylate tau at sites associated with Alzheimer disease. *J. Biol. Chem.* 288 23331–23347. 10.1074/jbc.M113.463984 23798682 PMC3743503

[B19] Chadli A. Felts S. J. Wang Q. Sullivan W. P. Botuyan M. V. Fauq A. (2010). Celastrol inhibits Hsp90 chaperoning of steroid receptors by inducing fibrillization of the Co-chaperone p23. *J. Biol. Chem.* 285 4224–4231. 10.1074/jbc.M109.081018 19996313 PMC2823561

[B20] Chang K. A. Kim H. S. Ha T. Y. Ha J. W. Shin K. Y. Jeong Y. H. (2006). Phosphorylation of amyloid precursor protein (APP) at Thr668 regulates the nuclear translocation of the APP intracellular domain and induces neurodegeneration. *Mol. Cell. Biol.* 26 4327–4338. 10.1128/mcb.02393-05 16705182 PMC1489099

[B21] Chen Y. Wang B. Liu D. Li J. J. Xue Y. Sakata K. (2014). Hsp90 chaperone inhibitor 17-AAG attenuates Abeta-induced synaptic toxicity and memory impairment. *J. Neurosci.* 34 2464–2470. 10.1523/JNEUROSCI.0151-13.2014 24523537 PMC3921421

[B22] Chin J. Palop J. J. Puolivali J. Massaro C. Bien-Ly N. Gerstein H. (2005). Fyn kinase induces synaptic and cognitive impairments in a transgenic mouse model of Alzheimer’s disease. *J. Neurosci.* 25 9694–9703. 10.1523/jneurosci.2980-05.2005 16237174 PMC6725734

[B23] Chin J. Palop J. J. Yu G. Q. Kojima N. Masliah E. Mucke L. (2004). Fyn kinase modulates synaptotoxicity, but not aberrant sprouting, in human amyloid precursor protein transgenic mice. *J. Neurosci.* 24 4692–4697. 10.1523/jneurosci.0277-04.2004 15140940 PMC6729387

[B24] Citri A. Gan J. Mosesson Y. Vereb G. Szollosi J. Yarden Y. (2004). Hsp90 restrains ErbB-2/HER2 signalling by limiting heterodimer formation. *EMBO Rep.* 5:1165. 10.1038/sj.embor.7400300 15568014 PMC1299195

[B25] Colin E. Régulier E. Perrin V. Dürr A. Brice A. Aebischer P. (2005). Akt is altered in an animal model of Huntington’s disease and in patients. *Eur. J. Neurosci.* 21 1478–1488. 10.1111/j.1460-9568.2005.03985.x 15845076

[B26] Cookson M. R. (2010). The role of leucine-rich repeat kinase 2 (LRRK2) in Parkinson's disease. *Nat. Rev. Neurosci.* 11:791. 10.1038/nrn2935 PMC4662256 21088684

[B27] Correas I. Díaz-Nido J. Avila J. (1992). Microtubule-associated protein tau is phosphorylated by protein kinase C on its tubulin binding domain. *J. Biol. Chem.* 267 15721–15728. 1639808

[B28] Coutadeur S. Benyamine H. Delalonde L. De Oliveira C. Leblond B. Foucourt A. (2015). A novel DYRK1A (Dual specificity tyrosine phosphorylation-regulated kinase 1A) inhibitor for the treatment of Alzheimer’s disease: effect on Tau and amyloid pathologies in vitro. *J. Neurochem.* 133 440–451. 10.1111/jnc.13018 25556849

[B29] Crevel G. Bates H. Huikeshoven H. Cotterill S. (2001). The Drosophila Dpit47 protein is a nuclear Hsp90 co-chaperone that interacts with DNA polymerase α. *J. Cell Sci.* 114 2015. 10.1242/jcs.114.11.2015 11493638

[B30] Csermely P. Schnaider T. SoTi C. Prohászka Z. Nardai G. (1998). The 90-kDa molecular chaperone family: structure, function, and clinical applications. *Compr. Rev. Pharmacol. Ther.* 79 129–168. 10.1016/s0163-7258(98)00013-8 9749880

[B31] Davis A. A. Leyns C. E. G. Holtzman D. M. (2018). Intercellular spread of protein aggregates in neurodegenerative disease. *Annu. Rev. Cell Dev. Biol.* 34 545–568. 10.1146/annurev-cellbio-100617-062636 30044648 PMC6350082

[B32] De Montigny A. Elhiri I. Allyson J. Cyr M. Massicotte G. (2013). NMDA reduces Tau phosphorylation in rat hippocampal slices by targeting NR2A receptors, GSK3β, and PKC activities. *Neural Plast.* 2013 261593–261593. 10.1155/2013/261593 24349798 PMC3856160

[B33] Dey B. Lightbody J. J. Boschelli F. (1996). CDC37 is required for p60v-src activity in yeast. *Mol. Biol. Cell* 7 1405–1417. 10.1091/mbc.7.9.1405 8885235 PMC275990

[B34] Dunn D. M. Woodford M. R. Truman A. W. Jensen S. M. Schulman J. Caza T. (2015). c-Abl Mediated Tyrosine Phosphorylation of Aha1 Activates Its Co-chaperone Function in Cancer Cells. *Cell Rep.* 12 1006–1018. 10.1016/j.celrep.2015.07.004 26235616 PMC4778718

[B35] Dushukyan N. Dunn D. M. Sager R. A. Woodford M. R. Loiselle D. R. Daneshvar M. (2017). Phosphorylation and ubiquitination regulate protein phosphatase 5 activity and its prosurvival role in kidney cancer. *Cell Rep.* 21 1883–1895. 10.1016/j.celrep.2017.10.074 29141220 PMC5699234

[B36] Eckl J. M. Scherr M. J. Freiburger L. Daake M. A. Sattler M. Richter K. (2015). Hsp90.C*dc*37 complexes with protein kinases form cooperatively with multiple distinct interaction sites. *J. Biol. Chem.* 290 30843–30854. 10.1074/jbc.M115.693150 26511315 PMC4692213

[B37] Estrada L. D. Chamorro D. Yanez M. J. Gonzalez M. Leal N. Von Bernhardi R. (2016). Reduction of blood amyloid-beta oligomers in alzheimer’s disease transgenic mice by c-Abl Kinase Inhibition. *J. Alzheimers Dis.* 54 1193–1205. 10.3233/jad-151087 27567806

[B38] Gandhi P. N. Chen S. G. Wilson-Delfosse A. L. (2009). Leucine-rich repeat kinase 2 (LRRK2): a key player in the pathogenesis of Parkinson’s disease. *J. Neurosci. Res.* 87 1283–1295. 10.1002/jnr.21949 19025767 PMC4072732

[B39] Gould C. M. Kannan N. Taylor S. S. Newton A. C. (2009). The chaperones Hsp90 and Cdc37 mediate the maturation and stabilization of protein kinase C through a conserved PXXP motif in the C-terminal tail. *J. Biol. Chem.* 284 4921–4935. 10.1074/jbc.M808436200 19091746 PMC2643500

[B40] Gray P. J. Jr. Stevenson M. A. Calderwood S. K. (2007). Targeting Cdc37 inhibits multiple signaling pathways and induces growth arrest in prostate cancer cells. *Cancer Res.* 67 11942–11950. 10.1158/0008-5472.can-07-3162 18089825

[B41] Hayes M. T. (2019). Parkinson’s Disease and parkinsonism. *Am. J. Med.* 132 802–807. 30890425 10.1016/j.amjmed.2019.03.001

[B42] Henstridge C. M. Hyman B. T. Spires-Jones T. L. (2019). Beyond the neuron-cellular interactions early in Alzheimer disease pathogenesis. *Nat. Rev. Neurosci.* 20 94–108. 10.1038/s41583-018-0113-1 30643230 PMC6545070

[B43] Hermida M. A. Dinesh Kumar J. Leslie N. R. (2017). GSK3 and its interactions with the PI3K/AKT/mTOR signalling network. *Adv. Biol. Regul.* 65 5–15. 10.1016/j.jbior.2017.06.003 28712664

[B44] Hinz M. Broemer M. Arslan S. C. Otto A. Mueller E.-C. Dettmer R. (2007). Signal responsiveness of IκB kinases is determined by Cdc37-assisted transient interaction with Hsp90. *J. Biol. Chem.* 282 32311–32319. 10.1074/jbc.m705785200 17728246

[B45] Humbert S. Bryson E. A. Cordelières F. P. Connors N. C. Datta S. R. Finkbeiner S. (2002). The IGF-1/Akt pathway is neuroprotective in huntington’s disease and involves huntingtin phosphorylation by akt. *Dev. Cell* 2 831–837. 10.1016/s1534-5807(02)00188-0 12062094

[B46] Isagawa T. Mukai H. Oishi K. Taniguchi T. Hasegawa H. Kawamata T. (2000). Dual effects of PKNα and protein kinase c on phosphorylation of tau protein by glycogen synthase Kinase-3β. *Biochem. Biophys. Res. Commun.* 273 209–212. 10.1006/bbrc.2000.2926 10873588

[B47] Jayapalan S. Natarajan J. (2013). The role of CDK5 and GSK3B kinases in hyperphosphorylation of microtubule associated protein tau (MAPT) in Alzheimer’s disease. *Bioinformation* 9 1023–1030. 10.6026/97320630091023 24497730 PMC3910359

[B48] Jiang J. Wang Z. H. Qu M. Gao D. Liu X. P. Zhu L. Q. (2015). Stimulation of EphB2 attenuates tau phosphorylation through PI3K/Akt-mediated inactivation of glycogen synthase kinase-3beta. *Sci. Rep.* 5:11765. 10.1038/srep11765 26119563 PMC4484244

[B49] Jin J. Tian R. Pasculescu A. Dai A. Y. Williton K. Taylor L. (2016). Mutational analysis of glycogen synthase kinase 3beta protein kinase together with kinome-wide binding and stability studies suggests context-dependent recognition of kinases by the chaperone heat shock protein 90. *Mol. Cell. Biol.* 36 1007–1018. 10.1128/MCB.01045-15 26755559 PMC4810477

[B50] Jinwal U. K. Abisambra J. F. Zhang J. Dharia S. O’leary J. C. Patel T. (2012). Cdc37/Hsp90 protein complex disruption triggers an autophagic clearance cascade for TDP-43 protein. *J. Biol. Chem.* 287 24814–24820. 10.1074/jbc.M112.367268 22674575 PMC3397908

[B51] Jinwal U. K. Trotter J. H. Abisambra J. F. Koren J. III Lawson L. Y. Vestal G. D. (2011). The Hsp90 kinase co-chaperone Cdc37 regulates tau stability and phosphorylation dynamics. *J. Biol. Chem.* 286 16976–16983. 10.1074/jbc.M110.182493 21367866 PMC3089541

[B52] Kang Q. Xiang Y. Li D. Liang J. Zhang X. Zhou F. (2017). MiR-124-3p attenuates hyperphosphorylation of Tau protein-induced apoptosis via caveolin-1-PI3K/Akt/GSK3β pathway in N2a/APP695swe cells. *Oncotarget* 8 24314–24326. 28186985 10.18632/oncotarget.15149 PMC5421849

[B53] Keramisanou D. Aboalroub A. Zhang Z. Liu W. Marshall D. Diviney A. (2016). Molecular mechanism of protein kinase recognition and sorting by the hsp90 kinome-specific cochaperone Cdc37. *Mol. Cell.* 62 260–271. 10.1016/j.molcel.2016.04.005 27105117 PMC4868553

[B54] Kimura Y. Rutherford S. L. Miyata Y. Yahara I. Freeman B. C. Yue L. (1997). Cdc37 is a molecular chaperone with specific functions in signal transduction. *Genes Dev.* 11 1775–1785. 10.1101/gad.11.14.1775 9242486

[B55] Kirouac L. Rajic A. J. Cribbs D. H. Padmanabhan J. (2017). Activation of Ras-ERK signaling and GSK-3 by amyloid precursor protein and amyloid beta facilitates neurodegeneration in Alzheimer’s Disease. *eNeuro* 4:ENEURO.0149-16.2017. 10.1523/ENEURO.0149-16.2017 28374012 PMC5367084

[B56] Klaić L. Morimoto R. I. Silverman R. B. (2012). Celastrol analogues as inducers of the heat shock response. Design and synthesis of affinity probes for the identification of protein targets. *ACS Chem. Biol.* 7 928–937. 10.1021/cb200539u 22380712 PMC3356480

[B57] La Spada A. R. Weydt P. Pineda V. V. (2011). “Frontiers in neuroscience huntington’s disease pathogenesis: mechanisms and pathways,” in *Neurobiology of Huntington’s Disease: Applications to Drug Discovery*, eds Lo D. C. Hughes R. E. (Boca Raton, FL: CRC Press).

[B58] Lackie R. E. Maciejewski A. Ostapchenko V. G. Marques-Lopes J. Choy W.-Y. Duennwald M. L. (2017). The Hsp70/Hsp90 chaperone machinery in neurodegenerative diseases. *Front. Neurosci.* 11:254. 10.3389/fnins.2017.00254 28559789 PMC5433227

[B59] Lau D. H. Hogseth M. Phillips E. C. O’neill M. J. Pooler A. M. Noble W. (2016). Critical residues involved in tau binding to fyn: implications for tau phosphorylation in Alzheimer’s disease. *Acta Neuropathol. Commun.* 4:49. 10.1186/s40478-016-0317-4 27193083 PMC4870772

[B60] Lazarevic V. Fieńko S. Andres-Alonso M. Anni D. Ivanova D. Montenegro-Venegas C. (2017). Physiological concentrations of amyloid beta regulate recycling of synaptic vesicles via alpha7 acetylcholine receptor and CDK5/Calcineurin signaling. *Front. Mol. Neurosci.* 10:221. 10.3389/fnmol.2017.00221 28785201 PMC5520466

[B61] Lee J. K. Kim N.-J. (2017). Recent advances in the inhibition of p38 MAPK as a potential strategy for the treatment of Alzheimer’s Disease. *Molecules* 22:1287. 10.3390/molecules22081287 28767069 PMC6152076

[B62] Li C. Götz J. (2017). Somatodendritic accumulation of Tau in Alzheimer’s disease is promoted by Fyn-mediated local protein translation. *EMBO J.* 36 3120–3138. 10.15252/embj.201797724 28864542 PMC5666608

[B63] Li D. Li C. Li L. Chen S. Wang L. Li Q. (2016). Natural product kongensin a is a non-canonical HSP90 inhibitor that blocks RIP3-dependent necroptosis. *Cell Chem. Biol.* 23 257–266. 10.1016/j.chembiol.2015.08.018 27028885

[B64] Li D. Xu T. Cao Y. Wang H. Li L. Chen S. (2015). A cytosolic heat shock protein 90 and cochaperone CDC37 complex is required for RIP3 activation during necroptosis. *Proc. Natl. Acad. Sci. U.S.A.* 112 5017–5022. 10.1073/pnas.1505244112 25852146 PMC4413296

[B65] Li X. Long J. He T. Belshaw R. Scott J. (2015). Integrated genomic approaches identify major pathways and upstream regulators in late onset Alzheimer’s disease. *Sci. Rep.* 5:12393. 10.1038/srep12393 26202100 PMC4511863

[B66] Li T. Chen X. Dai X. Y. Wei B. Weng Q. J. Chen X. (2017). Novel Hsp90 inhibitor platycodin D disrupts Hsp90/Cdc37 complex and enhances the anticancer effect of mTOR inhibitor. *Toxicol. Appl. Pharmacol.* 330 65–73. 10.1016/j.taap.2017.07.006 28711525

[B67] Lill C. M. (2016). Genetics of Parkinson’s disease. *Mol. Cell. Probes* 30 386–396. 10.1016/j.mcp.2016.11.001 27818248

[B68] Liu J. Yang J. Xu Y. Guo G. Cai L. Wu H. (2017). Roscovitine, a CDK5 inhibitor, alleviates sevoflurane-induced cognitive dysfunction via regulation Tau/GSK3β and ERK/PPARγ/CREB signaling. *Cell Physiol. Biochem.* 44 423–435. 10.1159/000485008 29141245

[B69] Liu X.-L. Xiao B. Yu Z.-C. Guo J.-C. Zhao Q.-C. Xu L. (1999). Down-regulation of Hsp90 could change cell cycle distribution and increase drug sensitivity of tumor cells. *World J. Gastroenterol.* 5 199–208. 11819430 10.3748/wjg.v5.i3.199 PMC4688469

[B70] Liu Y. Wang S. Ding D. Yu Z. Sun W. Wang Y. (2018). Up-regulation of Cdc37 contributes to schwann cell proliferation and migration after sciatic nerve crush. *Neurochem. Res.* 43 1182–1190. 10.1007/s11064-018-2535-6 29687307

[B71] Luo J. Benovic J. L. (2003). G protein-coupled receptor kinase interaction with Hsp90 mediates kinase maturation. *J. Biol. Chem.* 278 50908–50914. 10.1074/jbc.m307637200 14557268

[B72] Luo Q. Boczek E. E. Wang Q. Buchner J. Kaila V. R. I. (2017). Hsp90 dependence of a kinase is determined by its conformational landscape. *Sci. Rep.* 7 43996–43996. 10.1038/srep43996 28290541 PMC5349555

[B73] MacLean M. Picard D. (2003). Cdc37 goes beyond Hsp90 and kinases. *Cell Stress Chaperones* 8 114–119. 14627196 10.1379/1466-1268(2003)008<0114:cgbhak>2.0.co;2 PMC514862

[B74] Martin L. Latypova X. Wilson C. M. Magnaudeix A. Perrin M. L. Yardin C. (2013). Tau protein kinases: involvement in Alzheimer’s disease. *Ageing Res. Rev.* 12 289–309. 10.1016/j.arr.2012.06.003 22742992

[B75] Masters C. L. Bateman R. Blennow K. Rowe C. C. Sperling R. A. Cummings J. L. (2015). Alzheimer’s disease. *Nat. Rev. Dis. Primers* 1:15056. 10.1038/nrdp.2015.56 27188934

[B76] Mietelska-Porowska A. Wasik U. Goras M. Filipek A. Niewiadomska G. (2014). Tau protein modifications and interactions: their role in function and dysfunction. *Int. J. Mol. Sci.* 15 4671–4713. 10.3390/ijms15034671 24646911 PMC3975420

[B77] Millson S. H. Truman A. W. King V. Prodromou C. Pearl L. H. Piper P. W. (2005). A two-hybrid screen of the yeast proteome for Hsp90 interactors uncovers a novel Hsp90 chaperone requirement in the activity of a stress-activated mitogen-activated protein kinase, Slt2p (Mpk1p). *Eukaryot. Cell* 4 849–860. 10.1128/ec.4.5.849-860.2005 15879519 PMC1140089

[B78] Misra L. Mishra P. Pandey A. Sangwan R. S. Sangwan N. S. Tuli R. (2008). Withanolides from Withania somnifera roots. *Phytochemistry* 69 1000–1004. 10.1016/j.phytochem.2007.10.024 18061221

[B79] Mollapour M. Tsutsumi S. Truman A. W. Xu W. Vaughan C. K. Beebe K. (2011). Threonine 22 phosphorylation attenuates Hsp90 interaction with cochaperones and affects its chaperone activity. *Mol. Cell.* 41 672–681. 10.1016/j.molcel.2011.02.011 21419342 PMC3062913

[B80] Moriwaki Y. Kim Y.-J. Ido Y. Misawa H. Kawashima K. Endo S. (2008). L347P PINK1 mutant that fails to bind to Hsp90/Cdc37 chaperones is rapidly degraded in a proteasome-dependent manner. *Neurosci. Res.* 61 43–48. 10.1016/j.neures.2008.01.006 18359116

[B81] Moses M. A. Kim Y. S. Rivera-Marquez G. M. Oshima N. Watson M. J. Beebe K. E. (2018). Targeting the Hsp40/Hsp70 chaperone axis as a novel strategy to treat castration-resistant prostate cancer. *Cancer Res.* 78 4022–4035. 10.1158/0008-5472.CAN-17-3728 29764864 PMC6050126

[B82] Narayan M. Juan Z. Kaitlyn B. Chelsea G. Ashley Z. Daniel C. L. (2015). Withaferin A regulates LRRK2 levels by interfering with the Hsp90- Cdc37 chaperone complex. *Curr. Aging Sci.* 8 259–265. 10.2174/1874609808666150520111109 25989799

[B83] Neckers L. Blagg B. Haystead T. Trepel J. B. Whitesell L. Picard D. (2018). Methods to validate Hsp90 inhibitor specificity, to identify off-target effects, and to rethink approaches for further clinical development. *Cell Stress Chaperones* 23 467–482. 10.1007/s12192-018-0877-2 29392504 PMC6045531

[B84] Neckers L. Workman P. (2012). Hsp90 molecular chaperone inhibitors: are we there yet? *Clin. Cancer Res.* 18 64–76. 10.1158/1078-0432.CCR-11-1000 22215907 PMC3252205

[B85] Nika K. Soldani C. Salek M. Paster W. Gray A. Etzensperger R. (2010). Constitutively active Lck kinase in T cells drives antigen receptor signal transduction. *Immunity* 32 766–777. 10.1016/j.immuni.2010.05.011 20541955 PMC2996607

[B86] Noël A. Poitras I. Julien J. Petry F. R. Morin F. Charron J. (2015). ERK (MAPK) does not phosphorylate tau under physiological conditions in vivo or in vitro. *Neurobiol. Aging* 36 901–902. 10.1016/j.neurobiolaging.2014.11.005 25491074

[B87] Oberoi J. Dunn D. M. Woodford M. R. Mariotti L. Schulman J. Bourboulia D. (2016). Structural and functional basis of protein phosphatase 5 substrate specificity. *Proc. Natl. Acad. Sci. U.S.A.* 113 9009–9014. 10.1073/pnas.1603059113 27466404 PMC4987771

[B88] Ortega L. Calvillo M. Luna F. Perez-Severiano F. Rubio-Osornio M. Guevara J. (2014). 17-AAG improves cognitive process and increases heat shock protein response in a model lesion with Abeta25-35. *Neuropeptides* 48 221–232. 10.1016/j.npep.2014.04.006 24819277

[B89] Ota A. Wang Y. (2012). Cdc37/Hsp90 protein-mediated regulation of IRE1α protein activity in endoplasmic reticulum stress response and insulin synthesis in INS-1 cells. *J. Biol. Chem.* 287 6266–6274. 10.1074/jbc.M111.331264 22199355 PMC3307264

[B90] Park J. Choi H. Min J.-S. Kim B. Lee S.-R. Yun J. W. (2015). Loss of mitofusin 2 links beta-amyloid-mediated mitochondrial fragmentation and Cdk5-induced oxidative stress in neuron cells. *J. Neurochem.* 132 687–702. 10.1111/jnc.12984 25359615

[B91] Park J. S. Davis R. L. Sue C. M. (2018). Mitochondrial dysfunction in parkinson’s disease: new mechanistic insights and therapeutic perspectives. *Curr. Neurol. Neurosci. Rep.* 18:21. 10.1007/s11910-018-0829-3 29616350 PMC5882770

[B92] Peng B. Gu Y.-J. Wang Y. Cao F.-F. Zhang X. Zhang D.-H. (2016). Mutations Y493G and K546D in human HSP90 disrupt binding of celastrol and reduce interaction with Cdc37. *FEBS Open Bio* 6 729–734. 10.1002/2211-5463.12081 27398312 PMC4932452

[B93] Perez D. I. Gil C. Martinez A. (2011). Protein kinases CK1 and CK2 as new targets for neurodegenerative diseases. *Med. Res. Rev.* 31 924–954. 10.1002/med.20207 20577972

[B94] Phiel C. J. Wilson C. A. Lee V. M. Klein P. S. (2003). GSK-3alpha regulates production of Alzheimer’s disease amyloid-beta peptides. *Nature* 423 435–439. 10.1038/nature01640 12761548

[B95] Prodromou C. Roe S. M. O’brien R. Ladbury J. E. Piper P. W. Pearl L. H. (1997). Identification and structural characterization of the ATP/ADP-binding site in the Hsp90 Molecular chaperone. *Cell* 90 65–75. 10.1016/s0092-8674(00)80314-1 9230303

[B96] Reinhart P. H. Kaltenbach L. S. Essrich C. Dunn D. E. Eudailey J. A. Demarco C. T. (2011). Identification of anti-inflammatory targets for Huntington’s disease using a brain slice-based screening assay. *Neurobiol. Dis.* 43 248–256. 10.1016/j.nbd.2011.03.017 21458569 PMC3104027

[B97] Rezaei-Ghaleh N. Kumar S. Walter J. Zweckstetter M. (2016). Phosphorylation interferes with maturation of amyloid-beta fibrillar structure in the N terminus. *J. Biol. Chem.* 291 16059–16067. 10.1074/jbc.M116.728956 27252381 PMC4965556

[B98] Rosenstock T. R. Brett A. C. Rego A. C. (2012). “Neuronal pathways affected in Huntington’s Disease,” in *Multidisciplinary Viewpoint on Neurodegenerative Diseases*, ed. Di Carlo M. San Biagioand P. L. Bulone D. (Philadelphia PA: Research Signpost/Transworld Research Network), 1–26.

[B99] Rosenzweig R. Nillegoda N. B. Mayer M. P. Bukau B. (2019). The Hsp70 chaperone network. *Nat. Rev. Mol. Cell Biol.* 20 665–680. 10.1038/s41580-019-0133-3 31253954

[B100] Ryoo S. R. Jeong H. K. Radnaabazar C. Yoo J. J. Cho H. J. Lee H. W. (2007). DYRK1A-mediated hyperphosphorylation of Tau. A functional link between down syndrome and Alzheimer disease. *J. Biol. Chem.* 282 34850–34857. 10.1074/jbc.m707358200 17906291

[B101] Sahasrabudhe P. Rohrberg J. Biebl M. M. Rutz D. A. Buchner J. (2017). The Plasticity of the Hsp90 Co-chaperone system. *Mol. Cell.* 67:e945. 10.1016/j.molcel.2017.08.004 28890336

[B102] Saiki S. Sato S. Hattori N. (2012). Molecular pathogenesis of Parkinson’s disease: update. *J. Neurol. Neurosurg. Psychiatr.* 83 430–436. 10.1136/jnnp-2011-301205 22138181

[B103] Schapira A. H. Jenner P. (2011). Etiology and pathogenesis of Parkinson’s disease. *Mov. Disord.* 26 1049–1055. 21626550 10.1002/mds.23732

[B104] Schulte J. Littleton J. T. (2011). The biological function of the Huntingtin protein and its relevance to Huntington’s Disease pathology. *Curr. Trends Neurol.* 5 65–78. 22180703 PMC3237673

[B105] Seo H. R. Seo W. D. Pyun B.-J. Lee B. W. Jin Y. B. Park K. H. (2011). Radiosensitization by celastrol is mediated by modification of antioxidant thiol molecules. *Chem. Biol. Interact.* 193 34–42. 10.1016/j.cbi.2011.04.009 21570383

[B106] Seo J. Kritskiy O. Watson L. A. Barker S. J. Dey D. Raja W. K. (2017). Inhibition of p25/Cdk5 attenuates tauopathy in mouse and iPSC models of frontotemporal dementia. *J. Neurosci.* 37 9917–9924. 10.1523/JNEUROSCI.0621-17.2017 28912154 PMC5637118

[B107] Shi Y. Holtzman D. M. (2018). Interplay between innate immunity and Alzheimer disease: APOE and TREM2 in the spotlight. *Nat. Rev. Immunol.* 18 759–772. 10.1038/s41577-018-0051-1 30140051 PMC6425488

[B108] Siligardi G. Panaretou B. Meyer P. Singh S. Woolfson D. N. Piper P. W. (2002). Regulation of Hsp90 ATPase activity by the co-chaperone Cdc37p/p50 cdc37. *J. Biol. Chem.* 277 20151–20159. 10.1074/jbc.m201287200 11916974

[B109] Simic G. Babic Leko M. Wray S. Harrington C. Delalle I. Jovanov-Milosevic N. (2016). Tau protein hyperphosphorylation and aggregation in Alzheimer’s disease and other tauopathies, and possible neuroprotective strategies. *Biomolecules* 6:6. 10.3390/biom6010006 26751493 PMC4808800

[B110] Sittler A. Wanker E. E. Hartl F. U. Lueder G. Lehrach H. Priller J. (2001). Geldanamycin activates a heat shock response and inhibits huntingtin aggregation in a cell culture model of Huntington’s disease. *Hum. Mol. Genet.* 10 1307–1315. 10.1093/hmg/10.12.1307 11406612

[B111] Sluder I. T. Nitika N. Knighton L. E. Truman A. W. (2018). The Hsp70 co-chaperone Ydj1/HDJ2 regulates ribonucleotide reductase activity. *PLoS Genet.* 14:e1007462. 10.1371/journal.pgen.1007462 30452489 PMC6277125

[B112] Smith D. F. (1993). Dynamics of heat shock protein 90-progesterone receptor binding and the disactivation loop model for steroid receptor complexes. *Mol. Endocrinol.* 7 1418–1429. 10.1210/mend.7.11.7906860 7906860

[B113] Smith J. R. De Billy E. Hobbs S. Powers M. Prodromou C. Pearl L. (2015). Restricting direct interaction of CDC37 with HSP90 does not compromise chaperoning of client proteins. *Oncogene* 34 15–26. 10.1038/onc.2013.519 24292678 PMC3984902

[B114] Sonamoto R. Kii I. Koike Y. Sumida Y. Kato-Sumida T. Okuno Y. (2015). Identification of a DYRK1A inhibitor that induces degradation of the target kinase using co-chaperone CDC37 fused with Luciferase nanoKAZ. *Sci. Rep.* 5:12728. 10.1038/srep12728 26234946 PMC4522657

[B115] Stepanova L. Leng X. Parker S. B. Harper J. W. (1996). Mammalian p50Cdc37 is a protein kinase-targeting subunit of Hsp90 that binds and stabilizes Cdk4. *Genes Dev.* 10 1491–1502. 10.1101/gad.10.12.1491 8666233

[B116] Taipale M. Krykbaeva I. Koeva M. Kayatekin C. Westover K. D. Karras G. I. (2012). Quantitative analysis of HSP90-client interactions reveals principles of substrate recognition. *Cell* 150 987–1001. 10.1016/j.cell.2012.06.047 22939624 PMC3894786

[B117] Taylor S. S. Keshwani M. M. Steichen J. M. Kornev A. P. (2012). Evolution of the eukaryotic protein kinases as dynamic molecular switches. *Philos. Trans. R. Soc. Lond. B Biol. Sci.* 367 2517–2528. 10.1098/rstb.2012.0054 22889904 PMC3415842

[B118] Tripathi V. Darnauer S. Hartwig N. R. Obermann W. M. J. (2014). Aha1 can act as an autonomous chaperone to prevent aggregation of stressed proteins. *J. Biol. Chem.* 289 36220–36228. 10.1074/jbc.M114.590141 25378400 PMC4276884

[B119] Tsukahara F. Maru Y. (2010). Bag1 directly routes immature BCR-ABL for proteasomal degradation. *Blood* 116 3582–3592. 10.1182/blood-2009-10-249623 20675402

[B120] Vaughan C. K. Mollapour M. Smith J. R. Truman A. Hu B. Good V. M. (2008). Hsp90-dependent activation of protein kinases is regulated by chaperone-targeted dephosphorylation of Cdc37. *Mol. Cell.* 31 886–895. 10.1016/j.molcel.2008.07.021 18922470 PMC2568865

[B121] Verba K. A. Agard D. A. (2017). How Hsp90 and Cdc37 lubricate kinase molecular switches. *Trends Biochem. Sci.* 42 799–811. 10.1016/j.tibs.2017.07.002 28784328 PMC5621984

[B122] Verba K. A. Wang R. Y.-R. Arakawa A. Liu Y. Shirouzu M. Yokoyama S. (2016). Atomic structure of Hsp90-Cdc37-Cdk4 reveals that Hsp90 traps and stabilizes an unfolded kinase. *Science* 352 1542–1547. 10.1126/science.aaf5023 27339980 PMC5373496

[B123] Villemagne V. L. Fodero-Tavoletti M. T. Masters C. L. Rowe C. C. (2015). Tau imaging: early progress and future directions. *Lancet Neurol.* 14 114–124. 10.1016/S1474-4422(14)70252-2 25496902

[B124] Wang C. Y. Guo S. T. Wang J. Y. Yan X. G. Farrelly M. Zhang Y. Y. (2016). Reactivation of ERK and Akt confers resistance of mutant BRAF colon cancer cells to the HSP90 inhibitor AUY922. *Oncotarget* 7 49597–49610. 10.18632/oncotarget.10414 27391062 PMC5226532

[B125] Wang L. Cheng S. Yin Z. Xu C. Lu S. Hou J. (2015). Conditional inactivation of Akt three isoforms causes tau hyperphosphorylation in the brain. *Mol. Neurodegener.* 10 33–33. 10.1186/s13024-015-0030-y 26227811 PMC4521471

[B126] Wang L. Li L. Zhou Z. H. Jiang Z. Y. You Q. D. Xu X. L. (2017). Structure-based virtual screening and optimization of modulators targeting Hsp90-Cdc37 interaction. *Eur. J. Med. Chem.* 136 63–73. 10.1016/j.ejmech.2017.04.074 28482218

[B127] Wang L. Xie C. Greggio E. Parisiadou L. Shim H. Sun L. (2008). The Chaperone Activity of Heat Shock Protein 90 Is Critical for Maintaining the Stability of Leucine-Rich Repeat Kinase 2. *J. Neurosci.* 28 3384–3391. 10.1523/JNEUROSCI.0185-08.2008 18367605 PMC2564280

[B128] Weihofen A. Ostaszewski B. Selkoe D. J. Minami Y. (2007). Pink1 parkinson mutations, the Cdc37/Hsp90 chaperones and parkin all influence the maturation or subcellular distribution of Pink1. *Hum. Mol. Genet.* 17 602–616. 10.1093/hmg/ddm334 18003639

[B129] Westerheide S. D. Bosman J. D. Mbadugha B. N. A. Kawahara T. L. A. Matsumoto G. Kim S. (2004). Celastrols as inducers of the heat shock response and cytoprotection. *J. Biol. Chem.* 279 56053–56060. 10.1074/jbc.M409267200 15509580

[B130] Yang S. Pascual-Guiral S. Ponce R. Giménez-Llort L. Baltrons M. A. Arancio O. (2018). Reducing the levels of Akt activation by PDK1 knock-in mutation protects neuronal cultures against synthetic amyloid-beta peptides. *Front. Aging Neurosci.* 9:435. 10.3389/fnagi.2017.00435 29358916 PMC5766684

[B131] Yin X. Jin N. Shi J. Zhang Y. Wu Y. Gong C. X. (2017). Dyrk1A overexpression leads to increase of 3R-tau expression and cognitive deficits in Ts65Dn Down syndrome mice. *Sci. Rep.* 7:619. 10.1038/s41598-017-00682-y 28377597 PMC5428843

[B132] Yu Y. Hamza A. Zhang T. Gu M. Zou P. Newman B. (2010). Withaferin a targets heat shock protein 90 in pancreatic cancer cells. *Biochem. Pharmacol.* 79 542–551. 10.1016/j.bcp.2009.09.017 19769945 PMC2794909

[B133] Yun B. G. Matts R. L. (2005). Differential effects of Hsp90 inhibition on protein kinases regulating signal transduction pathways required for myoblast differentiation. *Exp. Cell Res.* 307 212–223. 10.1016/j.yexcr.2005.03.003 15922741

[B134] Zanphorlin L. M. Alves F. R. Ramos C. H. I. (2014). The effect of celastrol, a triterpene with antitumorigenic activity, on conformational and functional aspects of the human 90kDa heat shock protein Hsp90α, a chaperone implicated in the stabilization of the tumor phenotype. *Biochim. Biophys. Acta* 1840 3145–3152. 10.1016/j.bbagen.2014.06.008 24954307

[B135] Zhang H. Zhou C. Chen W. Xu Y. Shi Y. Wen Y. (2015). A dynamic view of ATP-coupled functioning cycle of Hsp90 N-terminal domain. *Sci. Rep.* 5 9542–9542. 10.1038/srep09542 25867902 PMC4394755

[B136] Zhang T. Li Y. Yu Y. Zou P. Jiang Y. Sun D. (2009). Characterization of Celastrol to Inhibit Hsp90 and Cdc37 Interaction. *J. Biol. Chem.* 284 35381–35389. 10.1074/jbc.M109.051532 19858214 PMC2790967

[B137] Zhang Y. Warnock G. L. Ao Z. Park Y. J. Safikhan N. Ghahary A. (2018). Amyloid formation reduces protein kinase B phosphorylation in primary islet β-cells which is improved by blocking IL-1β signaling. *PLoS One* 13:e0193184. 10.1371/journal.pone.0193184 29474443 PMC5825069

[B138] Zhang Y.-Q. Sarge K. D. (2007). Celastrol inhibits polyglutamine aggregation and toxicity though induction of the heat shock response. *J. Mol. Med.* 85 1421–1428. 10.1007/s00109-007-0251-9 17943263 PMC2262918

